# An Overlapping Case of Lupus Nephritis and IgG4-Related Kidney Disease

**DOI:** 10.14740/jocmr2189w

**Published:** 2015-05-08

**Authors:** Mazen Zaarour, Chanudi Weerasinghe, Ahmad Eter, Suzanne El-Sayegh, Elie El-Charabaty

**Affiliations:** a Department of Medicine, Staten Island University Hospital, Staten Island, NY, USA; b Department of Medicine, Division of Nephrology, Staten Island University Hospital, Staten Island, NY, USA

**Keywords:** IgG4-related kidney disease, Lupus nephritis, Tubulointerstitial nephritis, Membranous nephropathy, IgG4-related tubulointerstitial nephritis

## Abstract

We report a case of a 71-year-old Filipino female who was admitted to the hospital for abdominal pain, vomiting and diarrhea of 8 days duration. The patient was found to have marked acute kidney injury (AKI), which required hemodialysis in the next 3 days. Extensive workup revealed hematuria, subnephrotic range proteinuria, elevated anti-nuclear antibody (ANA) and elevated total immunoglobulin G (IgG) levels, with normal IgG4 and anti-dsDNA levels. On kidney biopsy, mild membranous glomerulonephritis was found, along with autoimmune tubulointerstitial nephritis (TIN) with a “full-house” pattern of immune deposits. These findings were suggestive of lupus interstitial nephritis. However, IgG4+ plasma cells were detected in the interstitium by immunostaining, favoring a diagnosis of IgG4-related kidney disease (IgG4-RKD). Our case highlights the difficulty in differentiating lupus nephritis (LN) from IgG4-RKD in some patients, raising the suspicion that these two entities can co-exist.

## Introduction

Systemic lupus erythematosus (SLE) is a chronic multi-organ disease with a variable clinical presentation. Kidney involvement is seen in half of these patients and remains the leading cause of death [1]. All renal compartments including glomerular, tubulointerstitial, and vascular components may be affected [2], and the renal biopsy remains essential for classification, prognosis and management of lupus nephritis (LN) [3].

Immunoglobulin G4-related kidney disease (IgG4-RKD) is a recently recognized disorder, the hallmark of which is dense lymphoplasmacytic infiltrate rich in IgG4+ plasma cells with interstitial fibrosis [4]. The most dominant feature of the disease is tubulointerstitial nephritis (TIN), although other glomerular lesions, such as membranous nephropathy, can be seen [4].

In most cases, the combination of clinical and serological features, supported by findings on kidney biopsy, is enough to establish a definitive diagnosis of either LN or IgG4-RKD. We report a challenging case where the complete distinction between these two entities was not possible, raising the suspicion of an overlap syndrome.

## Case Report

### Clinical history and initial laboratory data

A 71-year-old Filipino female presented to our hospital with abdominal pain, vomiting and diarrhea. The patient had been well until 8 days before admission, when the abdominal pain developed. The pain was diffuse, intermittent, and associated with episodes of vomiting and non-bloody diarrhea. Review of systems was negative except for minimal shortness of breath on exertion with no other respiratory symptoms. Prior medical history included hypertension treated with valsartan, hypothyroidism treated with levothyroxine, and thymoma resection. The patient was a non-smoker and had allergies to penicillin and amlodipine. Family history was non-contributory.

On admission, the patient’s body temperature was 99 °F, blood pressure was 128/78 mm Hg, and heart rate was 104/min. Physical exam was only remarkable for epigastric tenderness and mild bilateral lower extremity pitting edema. The patient was found to have marked leukocytosis of 44.0 × 10^9^/L (77% segmented neutrophils, 10% bands), along with acute kidney injury (AKI) with a blood urea nitrogen (BUN) of 88 mg/dL and a serum creatinine of 9.65 mg/dL (her baseline creatinine 2 years prior was 1.2 mg/dL). Hematologic findings were as follows: hemoglobin 11.7 g/dL, platelet count 255 × 10^9^/L, erythrocyte sedimentation rate (ESR) 61 mm/h. Aside from AKI and hypoalbuminemia, blood chemistry tests, including liver and pancreatic enzymes, were normal.

The patient denied any change in urine output or color, and no urinary symptoms. She also had no skin changes, oral ulcers, dry eyes or mouth, photosensitivity, arthralgias or upper respiratory symptoms. She reported no recent change in medications and no over the counter supplements.

Given the kidney injury, the valsartan was discontinued, intravenous fluids were started and a Foley catheter was placed to monitor her urine output. Analysis of the urine revealed blood with 12 - 20 red blood cells per high-power field, 6 - 12 white blood cells per high-power field and proteinuria of 2.6 g/day. Few white blood cells casts were seen with no red blood cells casts. A renal sonogram and a non-contrast abdominal computed tomography (CT) scan were inconclusive with normal kidney size (10.7 and 11.9 cm). A chest radiograph showed a right-sided upper lobe opacity. A chest CT confirmed the opacity and also demonstrated multiple small right-sided upper lobe granulomas of unclear etiology.

The admitting diagnosis was sepsis secondary to a presumed pneumonia, complicated by AKI, for which antibiotics were started. The kidney injury was initially attributed to severe pre-renal disease (from vomiting/diarrhea and sepsis) or acute tubular necrosis (ATN). The infectious workup was non-diagnostic, as evidenced by negative cultures (blood, stool, urine and later bronchoalveolar lavage smear for acid fast and cultures), quantiferon and urine streptococcal and legionella antigens.

The kidney function failed to improve with intravenous hydration (Table 1), and hemodialysis was initiated on day 4 for worsening uremia. Extensive workup for the kidney disease revealed elevated anti-nuclear antibody (ANA) titer, low-normal complement C4 level, reactive hepatitis B surface antigen (HBsAg), and elevated IgG levels. An immunoglobulin subclass analysis of peripheral blood revealed elevated levels of IgG1, IgG2, IgG3, along with normal IgG4 level. Additional studies were all negative (Table 2). Given the presence of hematuria, subnephrotic proteinuria, and worsening kidney function, a renal biopsy was scheduled.

**Table 1:** Evolution of BUN and Creatinine Over Days

Day	Admission	Day 2	Day 3	Day 4	Day 5	Day 6
BUN (mg/dL)	88	100	100	105	82	40
Creatinine (mg/dL)	9.65	9.27	9.20	9.70	9.0	5.80

**Table 2:** Laboratory Findings

Parameter	Value
Creatinine (mg/dL)	9.65
BUN (mg/dL)	88
Total protein (g/dL)	6.10
Albumin (g/dL)	2
Thyroid stimulating hormone (TSH) (µIU/mL)	0.81
Hepatitis B surface antigen (HbsAg)	Reactive
IgM hepatitis B core antibody	Non-reactive
Hepatitis B viral DNA (copies)	22576
Urinary protein (g/day)	2.60
ESR (mm/h)	61
Antinuclear antibody (ANA)	1:320, homogeneous
Antineutrophil cytoplasmic antibody (ANCA)	Negative
Complement C3 (mg/dL)	100 (reference range 80 - 180)
Complement C4 (mg/dL)	11 (reference range 10 - 45)
Anti-SSA antibody	Negative
Anti-SSB antibody	Negative
Anti-RNP antibody	Negative
Anti-dsDNA antibody	Negative
Smith antibody (anti-Sm)	Negative
IgG1 (mg/dL)	1230 (reference range 382 - 929)
IgG2 (mg/dL)	735 (reference range 241 - 700)
IgG3 (mg/dL)	418 (reference range 22 - 178)
IgG4 (mg/dL)	37.10 (reference range 4 - 86)
Serum protein electrophoresis (SPEP)	Normal
Glomerular basement membrane antibody (anti-GBM)	Negative
Cryoglobulin	Negative

### Kidney biopsy

**Figure 1:**
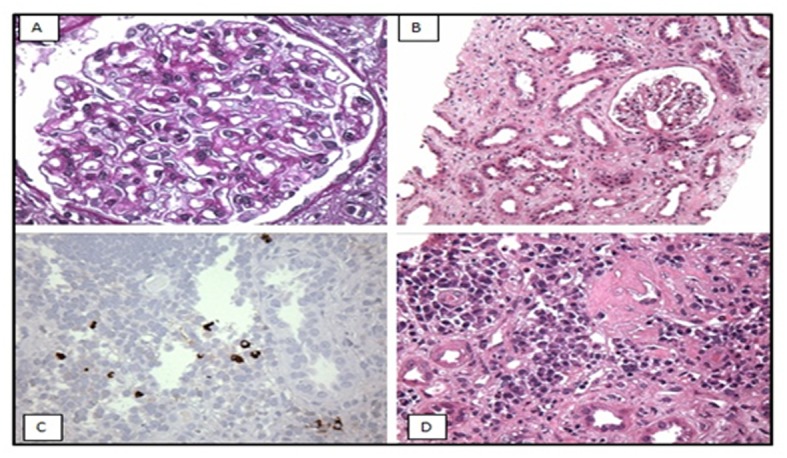
Light microscopy (A, B) shows: (A) mildly thickened glomerular basement membrane and (B) diffuse acute tubular injury with epithelial simplification consistent with acute tubular necrosis (ATN). Immunoperoxidase staining (C, D): immunostaining for IgG4 shows IgG4 positive plasma cells in the interstitium.

A CT-guided core needle biopsy of the right kidney was performed. The specimen submitted for light microscopy exhibited a total of 11 glomeruli, four of which were globally sclerotic. The remaining glomeruli appeared normal in size with patent capillaries. There was mild segmental to global increase in mesangial cell number and matrix. The glomerular basement membrane (GBM) appeared mildly thickened (Fig. 1A). Trichrome and silver stains delineated minute subepithelial fuchsinophilic deposits that focally indent the GBM, consistent with membranous features; however, no well-developed GBM spikes were seen. No glomeruli with neutrophil infiltration, overt endocapillary proliferation, necrotizing features or cellular crescents were identified. Virtually 100% of the cortex sampled had interstitial expansion by a mixture of fibrosis and edema associated with a patchy moderate inflammatory infiltrate of numerous plasma cells, lymphocytes, monocytes and occasional neutrophils. Tubular atrophy affected approximately 50% of the cortex sampled. The remaining tubules displayed diffuse acute injury with epithelial simplification (Fig. 1B), loss of brush border and enlarged reparative nuclei containing nucleoli. Many tubular basement membranes appeared thickened by PAS positive material. There also was mild arterio- and arteriolosclerosis, but no arteritis was identified.

Immunoperoxidase staining for IgG4 revealed up to 13 IgG4+ plasma cells per high-power field in the areas of most intense interstitial inflammation (Fig. 1C, D), suggestive of IgG4-related TIN. Immunostaining for phospholipase A_2_ receptor (PLA_2_R) antibody was negative.

**Figure 2:**
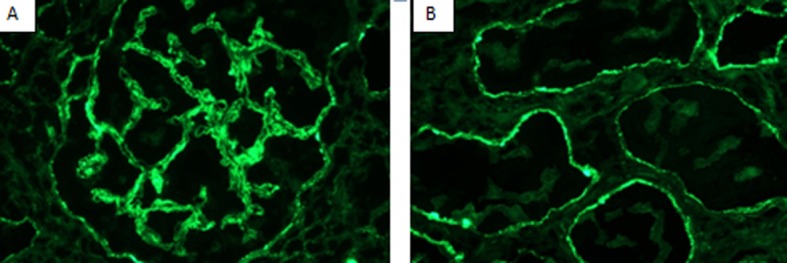
Immunofluorescence (IF) showing (A) granular glomerular capillary wall staining 2+ for IgG and (B) granular diffuse tubular basement membranes staining 2+ for IgG.

Immunofluorescence (IF) microscopy (Fig. 2) showed granular global mesangial and irregular capillary wall positivity in a predominantly sub-epithelial distribution. Abundant tubular basement membrane granular deposits also were found, staining for a “full-house” of immune reactants (Table 3), and suggestive of LN.

**Table 3:** Immunofluorescence Results

	Glomeruli	Tubules	Interstitium	Vessels
IgG	3 gloms 2+ Gran global mes Gran diffuse	TBM’s 2+ Sparse GCW	Neg	Neg
IgM	3 gloms 1+ Gran global mes Sparse GCW	TBM’s 1+ Gran focal	Plasma cells +1 cytoplasm	Neg
IgA	3 gloms 1+ Gran global mes Sparse GCW	TBM’s 1+ Gran focal	Plasma cells +1 cytoplasm	Neg
C3	3 gloms 1+ Gran global mes Sparse GCW	TBM’s 1+ Gran diffuse	Neg	Neg
C1	3 gloms neg	TBM’s 1+ Gran focal	Neg	Neg
FBGN	3 gloms neg	Neg	2+	Neg
ALB	3 gloms neg	Neg	Neg	Neg
KAPPA	3 gloms 2+ Gran global mes Sparse GCW	TBM’s 2+ Gran diffuse	Plasma cells +1 cytoplasm	Neg
LAMDA	3 gloms 2+ Gran global mes Sparse GCW	TBM’s 2+ Gran diffuse	Plasma cells +1 cytoplasm	Neg

Immunofluorescence results showing granular global mesangial and irregular capillary wall, as well as abundant tubular basement membrane granular deposits staining for a “full-house” of immune reactants (deposits containing IgM, IgA, IgG, Kappa, Lamda, C3 and C1). gloms: glomeruli; Gran: granular; mes: mesangial; GCW: glomerular capillary wall; TBM: tubular basement membrane; Neg: negative; FBGN: fibrinogen; ALB: albumin.

By electron microscopy, none of the two glomeruli sampled showed global or segmental sclerosis. The glomeruli displayed mild segmental increase in mesangial cell number and matrix containing small paramesangial electron dense deposits. The peripheral glomerular capillary walls were irregularly thickened by the presence of scattered small subepithelial and intramembranous deposits that range from electron dense to electron lucent, consistent with focal resorption. Overall, foot process effacement involved approximately 50% of the glomerular capillary surface area. No endothelial tubuloreticular inclusions (TRIs) were seen. Abundant granular electron dense deposits were seen involving the tubular basement membranes, associated with tubular atrophy, interstitial fibrosis, inflammation and edema.

In summary, the biopsy showed ATN, mild membranous glomerulonephritis, and severe autoimmune TIN with abundant tubular basement membrane deposits.

### Diagnosis

IgG4-related TIN with membranous nephropathy, and/or lupus membranous nephritis with TIN.

### Clinical follow-up

Although no definitive diagnosis was reached, the patient was started, the day after biopsy, on intravenous methylprednisone 1 g/day for 3 days, followed by prednisone 60 mg/day. She did not tolerate the glucocorticoid therapy and developed two episodes of gastrointestinal bleed, the second one severe enough to require distal gastrectomy. She was switched to mycophenolate mofetil (MMF) and was discharged home after a hospitalization of 33 days. She was seen on multiple occasions at the nephrology clinic and was successfully taken off hemodialysis 2 months later. After 5 months of immunosuppressive therapy, her creatinine level was 1.6 mg/dL and her proteinuria was 550 mg/day. More impressively, almost a year after her admission, her creatinine is currently back to her baseline level of 1.2 mg/dL and her proteinuria was as low as 427 mg/day. Complements, IgG4 and anti-dsDNA levels remained normal after 1 year. She also failed to show any clinical manifestation of SLE, aside from the nephropathy.

## Discussion

SLE is a chronic immune-mediated disease that can affect almost any organ system. Although overall mortality from this condition has decreased, nephropathy remains the leading cause of death [1]. The term “LN” is mainly used to refer to the immune complex-mediated glomerulonephritis [2]. The renal involvement in SLE has diverse morphologic manifestations with varying clinical presentations and consequences. The disease is staged according to the classification revised by the International Society of Nephrology (ISN) and the Renal Pathology Society (RPS) in 2003. This classification is based on light microscopy, IF, and electron microscopy findings from renal biopsy specimens [3].

IgG4-RKD is a fibro-inflammatory condition, the hallmark of which is dense lymphoplasmacytic infiltrates rich in IgG4+ plasma cells, storiform interstitial fibrosis and elevated serum IgG4 concentration [4]. It is part of a wider entity called IgG4-related disease (IgG4-RD), which is a well-defined systemic disease characterized by abundant IgG4+ plasma cell infiltration into the tissues [5]. The first recognized form of IgG4-RD was sclerosing pancreatitis, described in 1961 by Sarles et al [6], and later labeled as autoimmune pancreatitis (AIP). Since then, IgG4-RD has been described in nearly every organ system [5, 7, 8]. IgG4-RKD was first reported as a an extrapancreatic manifestation of AIP [9, 10], but recently it has been described in patients without pancreatitis [11, 12]. The most common pattern found on renal biopsy is IgG4-TIN [13, 14]. Glomerular lesions can also be seen, including membranous nephropathy (the most common glomerular lesion), membranoproliferative glomerulonephritis, and mesangial proliferative glomerulonephritis [4, 15-17].

The patient described in our case presented with marked kidney injury of unknown duration. The combination of microscopic hematuria and subnephrotic range proteinuria was suggestive of an underlying glomerular disease. An additional tubular involvement was suggested by the presence of white blood cells casts. The initial impression was that of an anti-neutrophil cytoplasmic antibody-associated (ANCA-associated) vasculitis, given the patient’s age and associated lung lesions. Additional pertinent serologic tests included a positive ANA, a low-normal complement C4 level, and an elevated total IgG level, all of which confirmed the suspicion of an autoimmune systemic process, but were non-specific for a particular disease. Given the rapid worsening of the kidney function, in the setting of a suspected vasculitis, the decision to perform a renal biopsy was made.

Kidney biopsy revealed mild membranous glomerulonephritis and severe autoimmune TIN, a combination of findings seen in both LN and IgG4-RKD. In the setting of an immune complex-mediated glomerulonephritis, some pathological findings on the biopsy strongly suggest a diagnosis of LN including 1) “full-house” IF staining for IgG, IgM, IgA, C3 and C1q; 2) extraglomerular immune deposits; 3) combined mesangial, subendothelial and subepithelial immune deposits and 4) the presence of endothelial TRIs [18, 19]. Our patient had all of these pathological findings, with the exception of the TRI, making the histological diagnosis of membranous lupus nephritis associated with severe lupus interstitial nephritis more likely than IgG4-RKD. On the other hand, immunostaining for IgG4 revealed up to 13 IgG4+ plasma cells per high-power field, favoring a diagnosis of IgG4-TIN. Immunostaining for phospholipase A_2_ receptor antibody, a marker of primary membranous glomerulopathy [20], was negative, labeling the membranous nephropathy as most likely due to a “secondary” injury. Furthermore, even though HBsAg was detected in the serum, the presence of auto-immune interstitial nephritis argues against the possibility of hepatitis B associated membranous nephritis. It is also likely that recent diarrhea and intravascular volume depletion have contributed to the acute tubular injury from ischemia, producing a superimposed ATN.

The diagnosis of SLE is based on a combination of clinical findings and laboratory evidence. The presence of four of the 11 American College of Rheumatology (ACR) criteria yields a sensitivity of 85% and a specificity of 95% for SLE [21]. When the Systemic Lupus International Collaborating Clinics (SLICC) group revised and validated the ACR SLE classification criteria in 2012, they classified a person as having SLE in the presence of biopsy-proven LN with ANA or anti-dsDNA antibodies or if four of the diagnostic criteria, including at least one clinical and one immunologic criterion, have been satisfied [22]. Our patient had only two SLICC diagnostic criteria (a positive ANA and renal involvement, and arguably a low-normal complement C4 level), insufficient to establish SLE as a definitive diagnosis. Elevated ANA and anti-dsDNA antibody titers along with depressed serum complement yield a combined sensitivity exceeding 90% for the diagnosis of SLE [23]. That being said, cases of lupus with a negative ANA/anti-dsDNA antibody and/or normal serum complements have been reported, suggesting that seronegativity does not exclude the diagnosis [24, 25]. Similarly, the absence of systemic findings of SLE does not exclude renal-limited lupus [26, 27]. The nephropathy in lupus includes a myriad of findings such as TIN and membranous nephropathy, both non-specific for the disease. However, the finding of a “full-house” pattern on IF favors the diagnosis of LN. In fact, the presence of “full-house” pattern, despite being described in non-lupus patients [28], makes LN very likely, even in the setting of a negative serology for SLE [29]. Furthermore, multiple reports described patients, mostly children, who presented initially with a “full-house” nephropathy, predicting the development of delayed clinical/serological SLE after a variable period of time [27, 30-32].

While in the past decade many studies have described IgG4-RKD, a proposal on diagnostic approach has been only recently published. In 2011, diagnostic criteria for IgG4-TIN and a diagnostic algorithm, using a set of diagnostic criteria for IgG4-RKD, were proposed by the group of North America [33] and the Japanese Society of Nephrology [34], respectively. According to the latter group, an elevated serum IgG4 level is required, making the diagnosis in our case “possible”, but not “definite”. On the other hand, our patient met the diagnostic criteria for IgG4-RKD set forth by the group of North America by having more than 10 IgG4+ plasma cells per high-power field (mandatory criterion), along with elevated total IgG levels. Radiographic features of the disease, such as low-attenuation renal lesions or diffuse marked enlargement of the kidneys [35], could not be adequately assessed given the non-contrast nature of the abdominal CT scan. Although other organ involvement was also not obvious, the presence of right-sided upper lobe granulomas can be part of IgG4-RD. Elevated total IgG level is prominent in many cases of IgG4-RKD, but the elevation in the level of IgG4 subclass is the most important finding, present in more than 90% of patients [36]. To our knowledge, only two cases were published with typical features of the disease but with normal serum IgG4 levels [37, 38], our case being the third described. Taken together, our patient could very likely have IgG4-RKD; however, an increase in the number of IgG4+ plasma cells was found to be non-specific for IgG4-RD. In fact, it may also be a feature of ANCA-associated vasculitis, granulomatosis with polyangitis, Churg-Strauss syndrome, lymphoproliferative disorders including malignant lymphoma, and even some inflammatory conditions [5, 39]. Moreover, Houghton et al have reported that IgG4+ plasma cell infiltration in the renal parenchyma may be evident even in some cases of diabetic nephropathy, idiopathic TIN, and even LN [39].

The mainstay of treatment for IgG4-RKD is glucocorticoids, with a dramatic and rapid response rate. There are no guidelines for the optimal dose and duration of treatment, and some patients might even relapse after discontinuation of immunosuppression [33]. Azathioprine, MMF, and methotrexate can be used as steroid-sparing drugs or to maintain remission. Similarly, a combination of prednisone and MMF is the standard-of-care treatment for severe LN [40, 41]. Although a definitive diagnosis of either LN or IgG4-RKD was not reached in our case, we opted to treat our patient with MMF, as it is a common therapeutic option for both entities and our patient could not tolerate steroids. Her response to treatment was a remarkable one. In fact, her serum creatinine was back to her baseline level, along with a marked reduction in the proteinuria.

In summary, we reported a challenging case where clinical, laboratory and even pathologic findings were inconclusive for a definitive diagnosis. The patient described had features of both LN and IgG4-RKD. The kidney biopsy findings of “full-house” tubular basement membrane deposits supported the possibility of lupus interstitial nephritis, correlating with the positive ANA. However, in the absence of clinical findings suggestive of SLE along with the near normal complements and anti-dsDNA levels, the differential diagnosis included IgG4-TIN. This possibility is further supported by the findings of IgG4+ plasma cells in the interstitium, despite the normal serum IgG4 level. A similar report was published in the literature describing a “possible” IgG4-TIN concomitant with membranous nephropathy, where the absence of “definite” diagnosis was attributed to the difficulty differentiating findings from membranous lupus nephritis with severe tubulointerstitial changes [42]. Our case confirms the numerous clinical and histopathological similarities between LN and IgG4-RKD, and raises the possibility of an overlap syndrome. Further studies are warranted to address this concern, as well as establish a clinical/histological “marker” able to provide a definitive diagnosis of either entity.
